# The Hide-and-Seek of Grain Boundaries from Moiré Pattern Fringe of Two-Dimensional Graphene

**DOI:** 10.1038/srep12508

**Published:** 2015-07-28

**Authors:** Jung Hwa Kim, Kwanpyo Kim, Zonghoon Lee

**Affiliations:** 1 School of Materials Science and Engineering, Ulsan National Institute of Science and Technology (UNIST), Ulsan 689-798, Korea; 2 Department of Physics, Ulsan National Institute of Science and Technology (UNIST), Ulsan 689-798, Korea

## Abstract

Grain boundaries (GBs) commonly exist in crystalline materials and affect various properties of materials. The facile identification of GBs is one of the significant requirements for systematical study of polycrystalline materials including recently emerging two-dimensional materials. Previous observations of GBs have been performed by various tools including high resolution transmission electron microscopy. However, a method to easily identify GBs, especially in the case of low-angle GBs, has not yet been well established. In this paper, we choose graphene bilayers with a GB as a model system and investigate the effects of interlayer rotations to the identification of GBs. We provide a critical condition between adjacent moiré fringe spacings, which determines the possibility of GB recognition. In addition, for monolayer graphene with a grain boundary, we demonstrate that low-angle GBs can be distinguished easily by inducing moiré patterns deliberately with an artificial reference overlay.

In the production of large-area two-dimensional (2D) graphene, grain boundaries (GBs) are inevitably produced^1^,^2^,^3^, which affect various properties of graphene, such as intrinsic tensile strength, fracture, failure, and electrical charge transport^4^,^5^,^6^,^7^,^8^,^9^,^10^,^11^,^12^. Recent studies have suggested that for bilayer graphene with single crystalline and polycrystalline layers stacked together, the layer with GBs dominates mechanical and electronic properties^13^,^14^,^15^. As a result, developing a method to easily identify GBs is a crucial requirement for studying polycrystalline graphene as well as other materials. In recent years, automatic identification of GBs was developed but the method still suffers from a high percentage of misidentification^16^. Transmission electron microscopy (TEM) mainly utilizes electron diffraction patterns (DP)^17^ and fast Fourier transform (FFT) of atomic resolution images^18^,^19^ to identify GBs. From DP and FFT, GBs can be estimated from compiling and marking the transition peaks of DPs, which is a tedious and time consuming method. In addition, these methods have serious limitations in the case of low angle GBs, because the adjacent peaks are shaded by the volume of peaks, which prevent the following of transition points.

We suggest a method to identify GBs directly on real-space images via moiré patterns in the bilayer graphene system. Moiré patterns are produced from superposition of equispaced parallel straight lines, as explained first by Rayleigh^20^,^21^. Moiré patterns can be used to amplify small distortions or displacements in lattice images^22^. As 2D layered materials have advanced as interesting systems^23^,^24^,^25^,^26^,^27^, moiré patterns have been researched extensively, due to the relationship between interesting characteristics and twist angles^28^. Depending on the interlayer rotation angle, the moiré pattern shows a distinct shape with different periodicity^29^,^30^,^31^,^32^,^33^,^34^,^35^,^36^, which is called moiré fringe spacing. In this paper, we elucidate the effects of interlayer rotation angle to the identification of GBs and provide the critical condition to determine whether GB recognition is possible or not. Furthermore, this paper proposes a method to distinguish GB readily by inducing moiré patterns with an artificial reference overlay. The reason why the moiré pattern is effective at distinguishing the GBs is also explained using a concept in visual perception.

## Results

**Figure 1:**
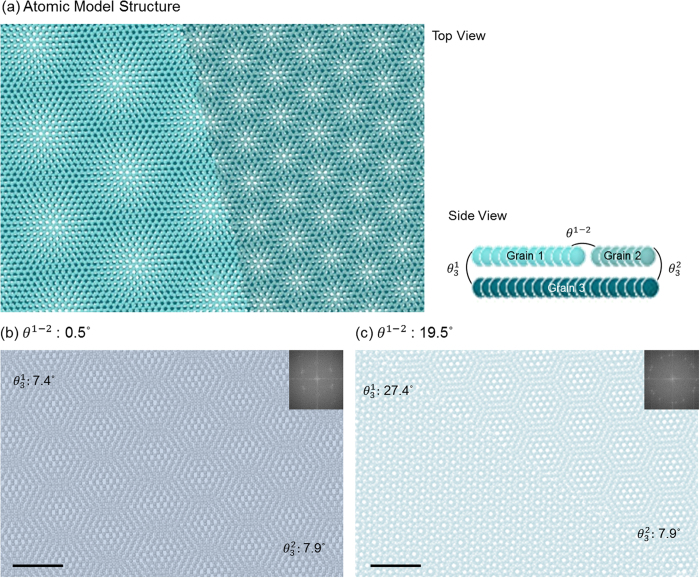
(**a**) An atomic model of bilayer graphene. Bottom layer is a single grain and top layer is composed of two tilt grains. The denoted angles represent rotational angles between grains. (**b**,**c**) Two atomic models with different *θ*^1−2^ value. The *θ*^1−2^ of (**b**) and (**c**) are 0.5° and 19.5°, respectively. The GB can be detected more readily with large *θ*^1−2^. Insets of (**b**) and (**c**) are FFTs of atomic models. Scale bar represents 2 nm.

Figure 1(a) shows a graphene bilayer with a GB in the upper layer (grain 1, 2) and a single crystal bottom layer (grain 3) as a schematic model. This structure includes the interlayer rotation angle 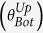 and in-plane rotation angle 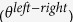. The interlayer rotation angle between grain 1 (grain 2) in the upper layer and grain 3 in the bottom layer is defined as 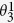 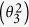. 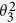 is also called the reference rotation angle for convenience. The in-plane rotation angle between grain 1 and grain 2 in the upper layer is defined as the intra-misorientation angle, written as *θ*^1−2^. The low *θ*^1−2^ case is also called as low angle GB.

Two distinct moiré patterns are formed when one layer including GB is overlaid on the other single-grain layer due to the difference of 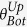 across the GB as shown in Fig. 1(b,c). The GB between two grains which have different crystallographic orientations results in a different *θ*^1−2^. Yuk *et al.* suggested that the GB in high *θ*^1−2^ is observed readily where two distinct moiré patterns join sharply^37^. On the other hand, in the case of low *θ*^1−2^, discrimination of superlattices is prevented due to the combined similar moiré patterns at the GB. As shown in Fig. 1(b,c), the extent of discrimination of the boundary is different due to the different *θ*^1−2^, that is, the difference between 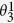 and 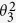. Figure 1(b) has a low *θ*^1−2^, 0.5°, which dissipates the trace of GB due to the mixing of similar moiré patterns at the GB, compared to Fig. 1(c) with a high *θ*^1−2^ of 19.5°. The FFT image, inset of Fig. 1(b), exhibits two sets of peaks which represent the bottom layer and upper layer, respectively. Two sets of peaks from grain 1 and grain 2 in the upper layer combined into a set of peaks which exhibit the upper layer, due to similar crystallographic orientation. The inset FFT image represents how difficult it is to distinguish two peaks, which is to say the crystallographic orientations of each grain, in the case of low *θ*^1−2^. On the other hand, Fig. 1(c) shows three sets of peaks which indicate grain 1, grain 2 and grain 3, respectively.

**Figure 2:**
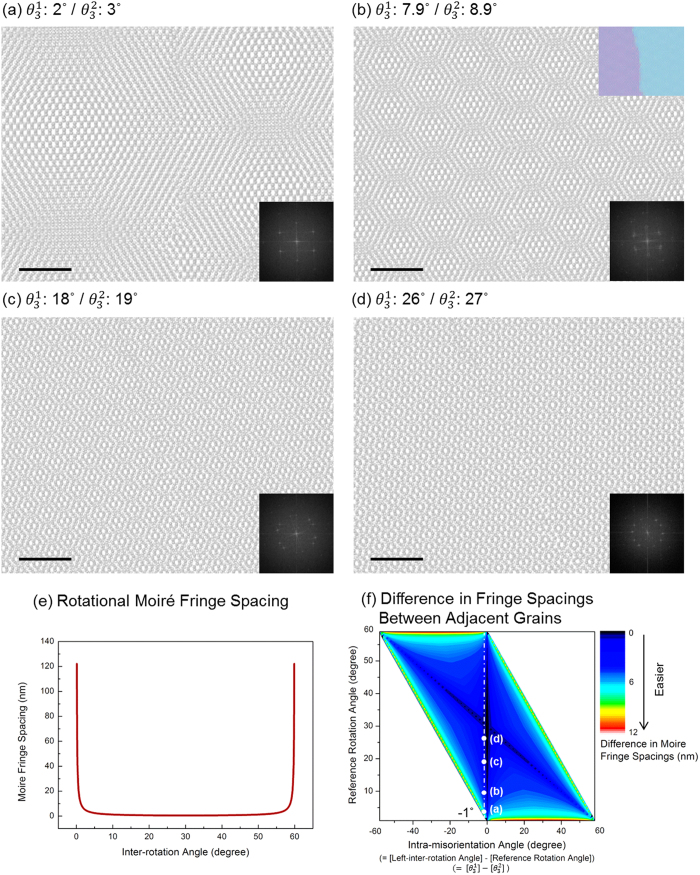
(**a**–**d**) Bilayer graphene atomic models showing various 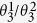 values with identical *θ*^1−2^. All models have *θ*^1−2^ = 1°. Reference rotation angles 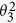 are (**a**) 3°, (**b**) 8.9°, (**c**) 19° and (**d**) 27°, respectively. When the intra-misorientation angle (*θ*^1−2^) are same, the detection of GB is more difficult when the *θ*^1−2^ goes to 0 or the sum of interlayer rotation angle 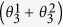 becomes 60°. Insets of (**a**–**d**) are FFTs of atomic models. Scale bar represents 2 nm. (**e**) Inter-rotation angle 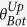 dependence of rotational moiré fringe spacings in graphene twisted bilayers. (**f**) Moiré fringe differences as a function of intra-misorientation angle and reference rotation angle. The GB distinguishability is shown in color. Red color represents the easier identification of GBs. The atomic models in panel (**a**-**d**) are marked. The observation of GB becomes more difficult from (**a**) to (**d**).

Although *θ*^1−2^ is low, 1° in Fig. 2(a–d), so the two peaks in the FFT of the image can’t be distinguished, the difference of the reference rotation angle 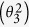 forms different superlattice domains shown as moiré patterns across the GB. Although they have the same low *θ*^1−2^ in Fig. 2(a–d), the vicinity of 30° of 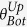 prevents the discrimination of GB, due to the mixing of similar moiré patterns previously suggested in Fig. 1(b,c). This means that the degree of GBs discrimination can be controlled by the 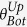, although the misorientation angle is the same. Mixing of similar moiré patterns means that the superlattice shapes on the left and right are similar. In detail, the periodicity of the superlattice, called moiré fringe spacing, is similar, because the shape of the superlattice depends on its periodicity. The periodicity of the moiré pattern in a bilayer homostructure like graphene-on-graphene depends only on 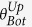, because the lattice parameters in each layer are exactly the same. Therefore, it is called rotational moiré fringe spacing and the expression for rotational moiré fringe spacing *d*_*rm*_ is
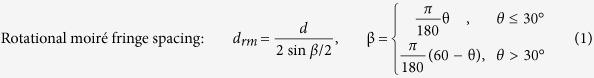
where *d* is the lattice plane spacing of graphene, which is 2.13 Å and β is the rotation angle between the upper and bottom layers 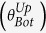 in radian. *θ* is rotation angle in degree, which has different condition at 30°.

As the graph of moiré fringe spacing versus 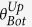
shows a negative exponential function shape from 0° to 30°, the spacing of the moiré fringe is large at low 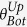 and decreases drastically as the 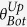 goes to 30°. In addition, due to the D_6_h symmetry of graphene, moiré fringe spacing graph is reflected at 30° as shown in Fig. 2(e). The results of Fig. 1(b,c) and Fig. 2(a–d) are related to the result of Fig. 2(e), which is the shape of rotational moiré fringe spacing. The graph of Fig. 2(f) shows the combined results of Fig. 1(b,c) and Fig. 2(a–d) using the difference in moiré fringe spacings between left and right moiré patterns. The x-axis represents the difference between 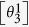 and 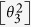 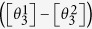. Therefore the magnitudes of positive and negative values on the x-axis represent *θ*^1−2^. The reason to use positive and negative values for the x-axis is to distinguish the two different cases. For example, when 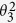 is 20° and *θ*^1−2^ is 5°, 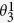 can be either 15° or 25°, which not only have different moiré fringe spacings but also different differences between the moiré fringe spacings of adjacent grains. The y-axis represents the reference rotation angle 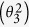, which shows the result of Fig. 2(a–d). Blue to red color presents the difference in moiré fringe spacings from 0, which means adjacent grains have same moiré fringe spacings, to 12 nm, which means the GBs are easier to detect. When the magnitude of *θ*^1−2^ approaches to 0° and the sum of 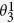 and 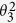 approaches 60°, the difference in moiré fringe spacings becomes lower (Fig. 2(e)), which goes to dark blue (Fig. 2(f)). For example, Fig. 2(a–d) are represented in white dots in Fig. 2(f), respectively. Figure 2(a) which is much easier to detect GB than any other cases in Fig. 2, is located near the red region, while Fig. 2(d) is near the dark blue region. It is obvious that the determination of GB using the moiré pattern is closely related to the difference of moiré fringe spacings.

**Figure 3:**
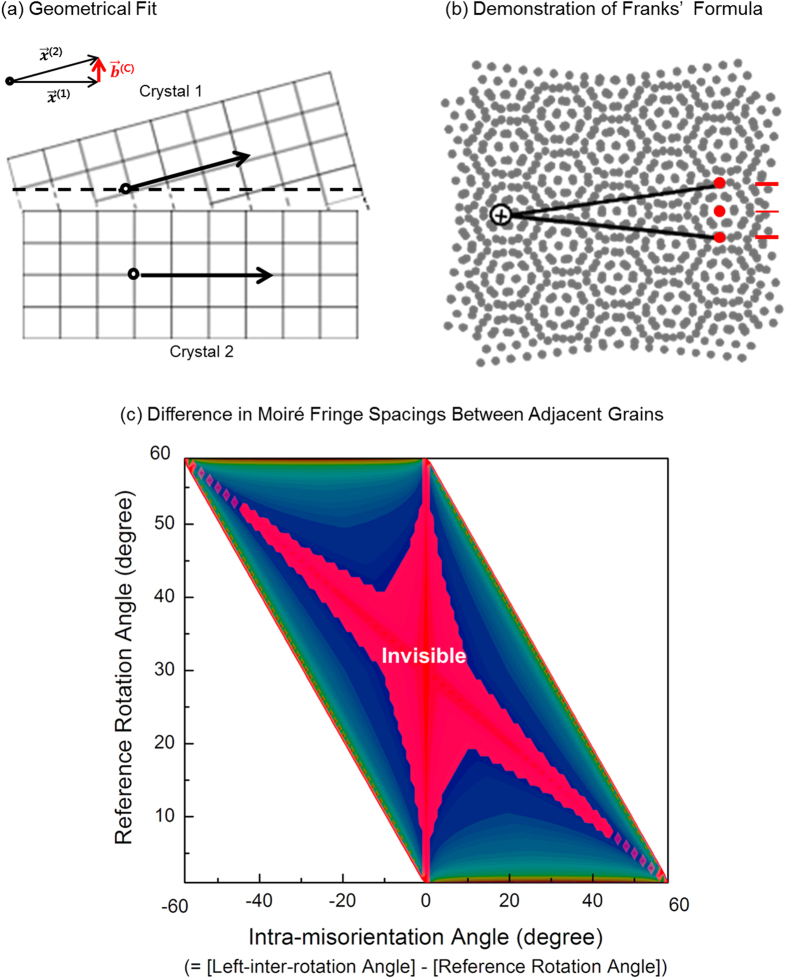
(**a**) Geometrical fit of two crystals. Crystals 1 and 2 are relatively rotated. (**b**) Demonstration of Frank’s formula with a hexagonal lattice. Difference vector can be described as the sum of discrete burgers vectors, which is represented in red. (**c**) Invisible GB region is shown as red color according to represented criterion.

The subgrain boundary is a network of discrete dislocations, shown as Fig. 3(a). When crystal 2, one of the divided parts, is rotated by angle θ around polar vector **θ**, while crystal 1 remains fixed,
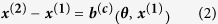
is satisfied where ***b***^(***c***)^ is the difference vector and ***x***^(**1**,**2**)^ are free vector in crystal 1 and 2, respectively^38^. Now let’s cut the two crystals, while maintaining the orientations, such that they can be placed on top of one another. Van der Waals interaction between graphene bilayers, which is thought of as no atomic forces between the two faces, forms a moiré pattern at the interface. The regions, where the crystal fit is perfect are represented as bright areas, while those where there is misfit are represented as dark areas. We call this “geometrical fit.” According to Frank’s formula, the function ***b***^(***c***)^ (***θ***, ***x***^(**1**)^) is approximately equal to the sum of the Burgers vectors of the dislocations crossed by ***x***. Figure 3(b) shows the moiré pattern from two overlaid hexagonal lattices. The difference vector from free vectors ***x***^(**1**)^ and ***x***^(**2**)^ is the sum of discrete burgers vectors, which are translation vectors of the crystal lattice, shown as discrete red lines in Fig. 3(b). Moiré fringe spacing depends on **θ**, and ***b***^(***c***)^ is the same way. From Frank’s formula, different moiré fringe spacings have different numbers of Burgers vectors. The magnitude of the Burgers vectors is the same as the lattice parameter of graphene, 0.213 nm. From this, we can’t distinguish the GBs when the difference of moiré fringe spacings is below the graphene lattice parameter, 0.213 nm.
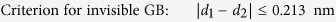
where *d*_1_ and *d*_2_ mean moiré fringe spacings in the region of grain 1 and grain 2, respectively. If we can’t recognize the unit lattice parameter from the difference of moiré fringe spacings, we can’t distinguish their difference, in terms of resolution. Surprisingly, this value coincides with the conventional resolution of TEM, which represents each lattice. Figure 3(b) shows the region of invisible GB, according to the difference in moiré fringe spacings, below 0.213 nm. The Fig. 2(c,d) case is included in this invisible region.

**Figure 4:**
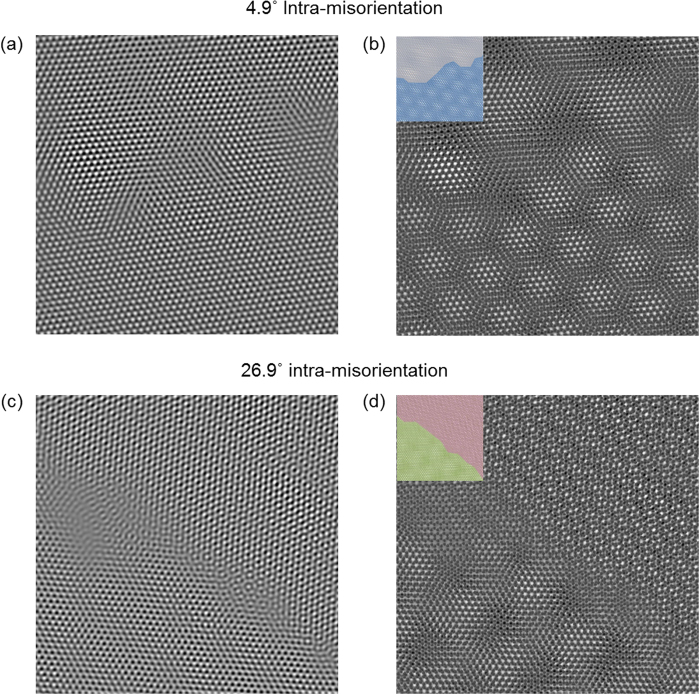
(**a**,**c**) The processed TEM images of graphene with two tilt grains. The images are obtained with inverse FFT of bilayer graphene. (**b**,**d**) The TEM images overlaid with reference layer. (**a**,**b**) have small *θ*^1−2^ and (**c**,**d**) have relative big *θ*^1−2^. Insets are color overlay images to show the grains more clearly.

The above results can be utilized by applying an artificial reference layer, mentioned by Hetherington and Dahmen^39^. The change of 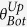 by rotating the artificial reference layer makes the GB obvious, although the *θ*^1−2^ is determined in the given TEM image. In this paper, inverse FFT of first diffracted peaks was used for selecting one layer with GB. Moiré patterns are formed from the overlaid artificial layer and the inverse FFT of the original TEM image. The difference of moiré fringe spacings varied from minimum to maximum, which means GB can be made obvious, by rotating the artificial reference layer. A bigger difference in moiré fringe spacings makes the detection of GB much easier. For example, there is a TEM image with a small angle GB, which is not shown evidently, as shown in Fig. 4(a). In contrast, the overlaid image in Fig. 4(b) demonstrates the GB can be observable by adjusting the rotation angle of the artificial overlay to make the proper difference of moiré fringe spacings. In the case shown in Fig. 4(c), although a GB in the TEM image is relatively observable, due to the high *θ*^1−2^, the overlaid image of Fig. 4(d) shows the GB even more distinguishable by increasing the difference of moiré fringe spacings. This application shows GBs intuitively on the image directly, not through a time-consuming and tedious process.

## Discussion

Using moiré patterns to distinguish the GBs is feasible and effective in terms of visual perception. Therefore we concentrated on the difference of moiré fringe spacings. In human eyes, each cell has size-selective receptive fields. In other words, certain receptive fields respond sensitively to one size. However the reactivity is not sensitive when the size is far from that size. For example, a receptive field which reacts to a large size is needed to recognize the large size of rods. When we gaze at one size, the receptive field tuned to that size is actively excited. The receptive fields tuned to similar size react a little. However, the receptive fields tuned far from that size don’t react to the stimulus. These reactivity patterns make us recognize that size. When we gaze at that size for a long time, the highly excited receptive field is not maintained anymore; this is called adaptation. Then if a smaller size is exposed, the adapted receptive field is insensitive relative to the opposite bigger size-selected receptive field. Therefore that smaller size is recognized as a far smaller, distorted size. This distortion is called the size after-effect^40^. When we see one moiré fringe spacing for some time, and then move to the other moiré fringe spacing, the distorted moiré fringe spacing is seen. In other words, bigger is overestimated and smaller is underestimated, due to the size after-effect. This effect is more effective when the difference of size between adjacent grains is small; this is called the distance paradox^40^. When we see similar moiré fringe spacings, the degree of distortion becomes bigger, which helps to detect GBs. However, according to the theory of size-selected increasing of threshold value, when we adjust to one size of pattern, the threshold value of contrast is almost 5-fold for a similar size of pattern, while there is no change of contrast threshold value for other shapes or sizes of pattern^41^. Therefore, in a low GB case, it is difficult to distinguish the GBs, although we see the moiré pattern.

**Figure 5:**
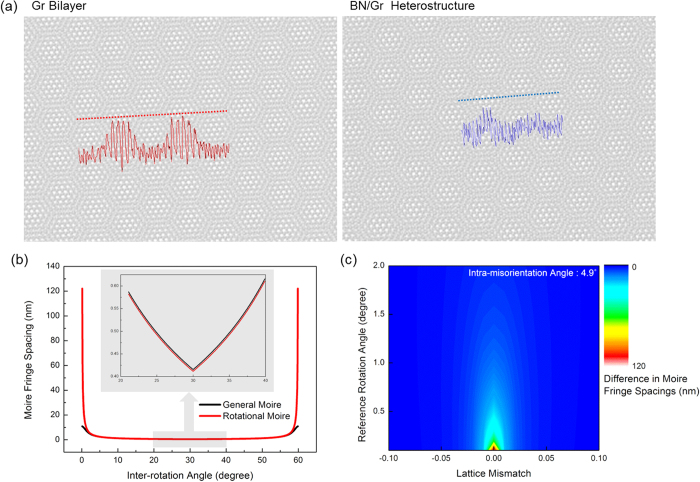
(**a**) Comparison of moiré patterns between homostructure and heterotructure. With a heterostructure (hexagonal boron nitride on graphene), the contrast modulation becomes low, as moiré pattern periodicity is not in line. (**b**) The comparison of rotational moiré fringe spacings and general moiré fringe spacings according to the 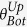, which are different whether lattice mismatch is 0 or 0.02 (BN/Gr bilayer). Inset image is magnified in near 30° of 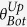. (**f**) The relationship of the lattice mismatch and moiré fringe difference. The grain distinguishability is more proper when using zero lattice mismatch reference layer.

In Fig. 4, the artificial reference layer with the same lattice as the two-dimensional material of the TEM image was applied to the TEM image. Sight is affected by spatial frequency and contrast^40^. However, in the case of BN/Graphene (Gr) heterostructure, which has a different lattice parameter, this structure makes it more difficult to detect GBs due to the ambiguous spatial frequency and contrast in Fig. 5(a). In addition, the difficulty in heterostructure can be explained by the difference of moiré fringe spacings. In Fig. 5(b), the spacing of rotational moiré fringe changes more drastically than that of general moiré fringe, which has 0.02 of lattice mismatch (in the case of BN/Gr heterostructure), in the range of low and the vicinity of 60° 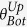. A general moiré pattern consists of a rotational moiré pattern and a translational moiré pattern, which is induced by the lattice mismatch between layers. The lattice mismatch between layers causes moiré fringe spacing even at 0 degrees of 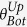; that is the reason for having a gradual slope in general moiré fringe spacing in low 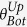. In detail, in Fig. 5(c), when the lattice mismatch is 0, the differences of moiré fringe spacings become maximum values at any 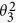, so that GB can be observed easily at zero lattice mismatch in terms of difference in moiré fringe spacings. At the vicinity of 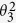, 30°, the difference moiré fringe spacings, which is related to the slope of moiré fringe spacing graph, is almost the same, which means the effect to observe GBs is similar in both rotational moiré pattern case and general moiré pattern case. Besides graphene, GBs in other two-dimensional materials can also be analyzed using an artificial overlay with an identical lattice.

In conclusion, we present why GB observation is difficult in low GB, in terms of the difference in moiré fringe spacings between superlattices which have different 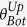. The lower the difference moiré fringe spacings exhibit, the harder it is to distinguish grains due to the combined moiré pattern. In addition, we can calculate the critical indistinguishable point according to Frank’s formula. From these results, GBs can be shown obviously by controlling 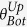, using a moiré pattern. Finally, we present an image to represent GBs obviously using an artificial reference layer to maximize the difference of moiré fringe spacings. This research is meaningful in that understanding and application of GB detection is established. It is useful even in low *θ*^1−2^ cases, which had difficulty in determining GBs using diffraction patterns and FFT. Our results will be useful for intrinsic GB studies, including phenomena and properties of GB, electronic structure modification, GB diffusion, and enhanced chemical reactivity at the boundary not only for single layers but also multilayers.

## Methods

### Simulation

The atomic model simulation images (Fig. 1,2 and Reference layer overlaid in Fig. 4(b,d)) were produced mainly by Jmol. Jmol is an open-source Java viewer for three-dimensional chemical structures. Jmol can read a variety of file types and output from quantum chemistry programs, and make animations of multi-frame files and computed normal modes from quantum programs. The main frame of the model was made by Chemsketch which is produced by ACD/Labs for free. TEM images and simulated images were overlaid using Adobe Photoshop.

### Sample preparation and characterization

The represented sample images are inversed FFT images of bilayer graphene including GB in one layer (Fig. 4(a,c)). Graphene was synthesized using chemical vapor deposition on a 25-μm thick copper substrate. After the copper foil was etched with a Na_2_S_2_O_9_ solution, bilayer graphene was obtained by successive direct transferring of monolayer graphene onto a Quantifoil TEM grid^37^. The TEM image was acquired using Cs image aberration-corrected FEI Titan Cubed TEM. The operating acceleration voltage was 80 kV. The Cs was fine tuned to obtain a third-order spherical aberration (C3) of −20 μm, which in consideration of the positive fifth-order aberration (C5) of 14.33 mm yielded optimal phase contrast with a slight positive defocus^37^.

## Additional Information

**How to cite this article**: Kim, J. H. *et al.* The Hide-and-Seek of Grain Boundaries from Moiré Pattern Fringe of Two-Dimensional Graphene. *Sci. Rep.* **5**, 12508; doi: 10.1038/srep12508 (2015).
